# Safety and tolerability of high-dose daily vitamin D_3_ supplementation in the vitamin D and type 2 diabetes (D2d) study—a randomized trial in persons with prediabetes

**DOI:** 10.1038/s41430-022-01068-8

**Published:** 2022-02-09

**Authors:** Karen C. Johnson, Anastassios G. Pittas, Karen L. Margolis, Anne L. Peters, Lawrence S. Phillips, Ellen M. Vickery, Jason Nelson, Patricia R. Sheehan, David Reboussin, Saul Malozowski, Ranee Chatterjee, D2d research group, Anastassios G. Pittas, Irwin Brodsky, Lisa Ceglia, Chhavi Chadha, Ranee Chatterjee, Bess Dawson-Hughes, Cyrus Desouza, Rowena Dolor, John Foreyt, Adline Ghazi, Daniel S. Hsia, Karen C. Johnson, Sangeeta R. Kashyap, Sun Kim, Erin S. LeBlanc, Michael R. Lewis, Emilia Liao, Saul Malozowski, Lisa M. Neff, Patrick O’Neil, Jean Park, Anne Peters, Lawrence S. Phillips, Richard Pratley, Philip Raskin, Neda Rasouli, David Robbins, Clifford Rosen, Vanita R. Aroda, Patricia Sheehan, Myrlene A. Staten, James H. Ware, William C. Knowler

**Affiliations:** 1 Department of Preventive Medicine, University of Tennessee Health Science Center, Memphis, TN USA; 2 Division of Endocrinology, Diabetes, and Metabolism, Tufts Medical Center, Boston, MA USA; 3 HealthPartners Institute, Minneapolis, MN USA; 4 Keck School of Medicine, University of Southern California, Los Angeles, CA USA; 5 Atlanta VA Medical Center and Emory University School of Medicine, Decatur GA and Atlanta, GA USA; 6 Tufts Clinical and Translational Science Institute, Biostatistics, Epidemiology, and Research Design Center, Tufts Medical Center, Boston, MA USA; 7 Spaulding Rehabilitation Hospital, Boston, MA USA; 8 Department of Biostatistics and Data Science, Wake Forest School of Medicine, Winston-Salem, NC USA; 9 National Institute of Diabetes and Digestive and Kidney Disease, National Institutes of Health, Bethesda, MD USA; 10 Department of Medicine, Duke University School of Medicine, Durham, NC USA; 11 Tufts Medical Center, Boston, MA USA; 12 Maine Medical Center Research Institute, Scarborough, ME USA; 13 Tufts Medical Center, Boston, MA USA; 14 Health Partners Research Foundation, Minneapolis, MN USA; 15 Duke University Medical Center, Durham, NC USA; 16 Jean Mayer USDA Human Nutrition Research Center on Aging at Tufts University, Boston, MA USA; 17 Omaha VA Medical Center, University of Nebraska Medical Center, Omaha, NE USA; 18 Baylor College of Medicine, Houston, TX USA; 19 MedStar Good Samaritan Hospital, Baltimore, MD USA; 20 Pennington Biomedical Research Center, Baton Rouge, LA USA; 21 University of Tennessee Health Science Center, Memphis, TN USA; 22 Cleveland Clinic, Cleveland, OH USA; 23 Stanford University Medical Center, Stanford, CA USA; 24 Kaiser Permanente Center for Health Research NW, Portland, OR USA; 25 University of Vermont–Central Laboratory, Burlington, VT USA; 26 Northwell Health Lenox Hill Hospital, New York, NY USA; 27 National Institute of Diabetes and Digestive and Kidney Diseases, Bethesda, MD USA; 28 Northwestern Memorial Hospital, Chicago, IL USA; 29 Medical University of South Carolina, Charleston, SC USA; 30 MedStar Health Research Institute, Hyattsville, MD USA; 31 Keck School of Medicine of the University of Southern California, Los Angeles, CA USA; 32 Atlanta VA Medical Center, Decatur, GA and Emory University School of Medicine, Atlanta, GA USA; 33 AdventHealth Translational Research Institute for Metabolism and Diabetes, Orlando, FL USA; 34 University of Texas Southwestern Medical Center, Dallas, TX USA; 35 University of Colorado, School of Medicine and VA Eastern Colorado Health Care System, Aurora, CO USA; 36 University of Kansas Medical Center, Kansas City, KS USA; 37 Brigham and Women’s Hospital, Boston, MA USA; 38 Spaulding Rehabilitation Network, Boston, MA USA; 39 Harvard T.H. Chan School of Public Health, Boston, MA USA; 40 National Institute of Diabetes and Digestive and Kidney Diseases, Phoenix, AZ USA

**Keywords:** Nutrition, Metabolic disorders

## Abstract

**Background/Objectives:**

Routine use of vitamin D supplements has increased substantially in the United States. However, the safety and tolerability of long-term use of high-dose vitamin D are not known. We assessed the safety and tolerability of high-dose, daily vitamin D_3_ in the vitamin D and type 2 diabetes (D2d) study.

**Subjects/Methods:**

In total, 2423 overweight/obese persons with prediabetes were randomized in a double-blind manner to either 4000 IU of vitamin D_3_ (the tolerable upper intake level for adults by the National Academy of Medicine) taken daily or matching placebo. All participants were included in this analysis. Incident adverse events (AE) were ascertained 4 times a year at in-person visits (twice a year) and interim remote encounters (twice a year) and were defined as untoward or unfavorable medical occurrences. Serious adverse events (SAE) included death, life-threatening events, and hospitalizations.

**Results:**

A total of 8304 AEs occurred during 3 years of follow-up and were less frequent in the vitamin D group compared to placebo (Incidence Rate Ratio [IRR] = 0.94; 95% Confidence Interval (CI) 0.90, 0.98). The overall frequency of protocol-specified AEs of interest, which included nephrolithiasis, hypercalcemia, hypercalciuria, or low estimated glomerular filtration rate, was low and did not differ by group. There were no significant between-group differences in total SAEs (IRR = 0.96 (0.81, 1.14)).

**Conclusion:**

Vitamin D_3_ supplementation at 4000 IU per day was safe and well tolerated among overweight/obese participants at high risk for diabetes who were appropriately monitored for safety. In this population, this dose of vitamin D_3_ did not increase risk of AEs or SAEs, including those previously associated with vitamin D such as hypercalcemia, hypercalciuria, or nephrolithiasis.

**Clinical Trial Registration:**

ClinicalTrials.gov NCT01942694, prospectively registered September 16, 2013

## Introduction

There has been substantial interest in vitamin D and its potential role in prevention of a number of chronic diseases, including diabetes, cardiovascular disease, and cancer [1–3]. Recently, the focus of vitamin D research has turned to randomized controlled trials of vitamin D at doses higher than typically recommended compared to placebo in persons who were generally considered to be vitamin D sufficient by current guidelines [4–9]. However, there is insufficient evidence regarding the safety and tolerability of vitamin D supplementation at these higher doses [10, 11]. At the same time, routine use of vitamin D supplements, especially at doses higher than are typically recommended in guidelines, has increased substantially in the United States, despite insufficient data regarding potential safety issues or side effects with longer term use at these higher doses [12].

The Vitamin D and Type 2 Diabetes (D2d) study was a randomized clinical trial of vitamin D_3_ supplementation at a dose of 4000 IU per day compared to placebo among overweight/obese participants who were at high risk for type 2 diabetes [6]. In this pre-specified analysis, we examined the safety and tolerability of vitamin D_3_ supplementation in the D2d study, which tested the vitamin D dose that is considered the tolerable upper intake level (UL) for adults by the National Academy of Medicine [13].

## Subjects and methods

### Trial design overview

The D2d study was a randomized, double-blind, placebo-controlled clinical trial conducted to evaluate the safety and efficacy of oral vitamin D_3_ for diabetes prevention in adults at high risk for type 2 diabetes [5]. The study involved collaboration among 22 academic medical centers in the United States (d2dstudy.org/sites) [5]. The trial protocol is available at D2dstudy.org. A sponsor-appointed data and safety monitoring board approved the protocol and provided independent study monitoring. The institutional review board at each clinical site also approved the protocol, and all participants provided written informed consent. The study was conducted in accordance with the principles of the Declaration of Helsinki and Good Clinical Practice. The statistical team at the D2d coordinating center (Division of Endocrinology, Tufts Medical Center, Boston, USA) performed the statistical analysis and vouches for its accuracy.

### Participants

Eligible participants met at least two of three glycemic criteria for prediabetes as defined by the 2010 American Diabetes Association (ADA) guidelines. Other inclusion criteria were age greater than or equal to 30 years (25 years for American Indians, Alaska Natives, Native Hawaiians, or other Pacific Islanders) and body mass index (BMI) of 24–42 kg/m^2^ (22.5–42 kg/m2 for Asian Americans) [14]. A low serum 25 hydroxyvitamin D (25[OH]D) concentration was not an inclusion criterion. Key exclusion criteria included use of diabetes or weight-loss medications or a history of hyperparathyroidism, nephrolithiasis, hypercalcemia, chronic kidney disease (defined as estimated glomerular filtration rate [eGFR] <50 mL/min/1.73 m^2^), calcium-to-creatinine ratio greater than 0.275 at baseline, or bariatric surgery. Persons were also excluded for use of supplements containing total doses of vitamin D higher than 1000 IU/day or total calcium higher than 600 mg/day. A complete list of exclusion criteria has been published and the recruitment process described previously [5, 6, 15].

### Intervention and Procedures

Participants were randomized in a 1:1 allocation ratio to take once-daily, a soft-gel containing either 4000 IU of vitamin D_3_ (cholecalciferol) or matching placebo, with stratification by site, BMI (<30 or ≥30 kg/m^2^), and race (White or non-White). Participants and all study staff were blinded to treatment assignment. Participants were asked to limit the use of outside-of-study vitamin D to 1000 IU per day from all supplements. To optimize safety, participants were also asked to limit calcium supplements to 600 mg per day.

In-person follow-up visits occurred at month 3, month 6, and twice per year thereafter. Midway between the in-person visits starting after month 6, an interim contact (phone or email) took place. All visits and contacts were designed to promote retention, encourage adherence, and assess for diabetes diagnosis outside of the study, tolerability of study pills, occurrence of adverse events, and personal use of vitamin D supplements higher than allowed by study protocol. Participants were monitored for adverse events (AE) including those previously associated with vitamin D supplementation, and incident AEs were ascertained at visits and interim encounters 4 times a year in a similar manner in both groups. The protocol outlined in detail the safety parameters for which the study trial pills should be discontinued (e.g., nephrolithiasis, hypercalcemia, low eGFR, etc.) [6].

Vitamin D content of study trial pills was analyzed for each production lot at bottling for the vitamin D_3_ pills as well as for placebo pills to confirm they were free of vitamin D. Acceptable vitamin D_3_ content for the active vitamin D pill was pre-defined as 80–120% of the 4000 IU planned dosage.

### Outcomes

The primary outcome of the D2d study was time to incident diabetes [14]. Participants who met the primary outcome of diabetes remained on the study pills and continued to be followed for safety and additional outcomes. The primary results have been previously published [6].

Adverse events were ascertained at each participant contact by study staff. At these encounters, each participant was asked if they had experienced any changes to their health or had sought medical care since last contact. If the participant responded affirmatively, study staff collected information on the health change or reason for and timing of medical care including diagnostic tests, diagnosis, and treatment. Study staff also reviewed with participants previously reported ongoing AEs to determine if the event had resolved.

An AE was defined as any untoward or unfavorable and unintended medical occurrence (including symptom, physical sign, laboratory finding, or disease) observed in or experienced by a participant, whether or not it was considered study related. A serious adverse event (SAE) was defined as any AE that resulted in death, a life-threatening event, a new inpatient hospitalization or prolongation of an existing hospitalization, a persistent or significant disability or incapacity, a congenital anomaly or birth defect, or any other significant hazard that, based upon appropriate medical judgment by the investigators, may have jeopardized the participant’s health and may have required medical or surgical intervention to prevent one of the outcomes listed in this definition. For all SAEs reported by the sites, medical records were collected and reviewed by the study’s Safety and Outcomes Subcommittee, composed of D2d investigators who were blinded to the participant’s assignment.

Key protocol-specified AEs of interest included hypercalcemia, hypercalciuria, low eGFR and nephrolithiasis. Follow-up serum calcium (assessed at each site’s local laboratory), the urine calcium-to-creatinine ratio (assessed at the central laboratory), and serum creatinine (assessed at each site’s local laboratory) to estimate GFR (calculated centrally) were measured at month 3, and annually thereafter. If serum calcium value (uncorrected for albumin concentration) was greater than the site’s clinical laboratory upper level of normal and less than or equal to the upper level of normal plus 1 mg/dL, participants were queried about calcium intake and supplements and medications (e.g., use of hydrochlorothiazide) and educated about the use of calcium supplements. Testing was repeated within 6 weeks. If the repeat serum calcium value was greater than the site’s clinical laboratory upper level of normal, the participant was confirmed to have met the outcome of hypercalcemia; study pills were stopped, and the participant was referred to their health care provider. If the first measurement of calcium was greater than the site’s clinical laboratory upper level of normal plus 1 mg/dL, no repeat testing was required and the participant was considered to have met the outcome of hypercalcemia; study pills were stopped, and the participant was referred to their health care provider. Regarding the adverse event of low eGFR, if eGFR value was greater than 30 and less than 40 mL/min/1.73m^2^, testing was repeated within 4 weeks. If repeat eGFR was equal to or less than 30 mL/min/1.73m^2^, the participant was confirmed to have met the outcome of low eGFR, study pills were stopped, and the participant was referred to their health care provider. If the first eGFR measurement was equal to or less than 30 mL/min/1.73m^2^, then no repeat testing was required, and the participant was considered to have met the outcome of low eGFR; study pills were stopped, and the participant was referred to their health care provider. Regarding the adverse event of hypercalciuria, if urine calcium-to-creatinine ratio was greater than 0.375, participants were queried about calcium intake and supplements, and testing was repeated within 4 weeks. If repeat urine calcium-to-creatinine ratio remained greater than 0.375, then the participant was considered to have met the outcome of hypercalciuria; study pills were stopped, and the participant was referred to their health care provider. The 0.375 cutoff was chosen because it represents the calcium-to-creatinine ratio in a random spot urine specimen that corresponds to a 24-hour urine calcium of 400 mg/gram, which is the upper reference range for men.

Participants were asked to contact site staff to report the occurrence of a kidney stone and were additionally specifically queried about kidney stones at each contact (phone or in-person visit). All reports of kidney stone were included in the nephrolithiasis outcome and participants reporting a kidney stone were instructed to discontinue study pills. If available, medical records related to nephrolithiasis were collected and then adjudicated by the study’s safety and outcomes subcommittee. For all of the above key protocol-specified AEs of interest where study pills were stopped per protocol, the pills were discontinued without unmasking participants or study staff, and participants continued in the study and completed all subsequent planned visits and measurements including collection of the primary outcome and safety assessment.

The D2d study did not specifically query the participants regarding falls using a validated questionnaire, but injuries and musculoskeletal events were self-reported by participants and were included in the overall assessment of safety.

Additional protocol-specified AEs of interest reported in this analysis were potentially related to the study pills and included polyuria, nausea, vomiting, poor appetite, metallic taste, hyperphosphatemia, anemia, weakness, fatigue, insomnia, and headache (all self-reported).

Participants could request to discontinue study pills at any point and for any reason. Participants who discontinued the study pills regardless of reason (AE or personal choice) were followed for the efficacy outcome per intent-to-treat principle.

### Laboratory testing

Serum calcium and creatinine were analyzed locally at each site, and eGFR was calculated centrally using the Chronic Kidney Disease Epidemiology Collaboration (CKD-EPI) equation adjusted for race, as the formula was commonly applied when the study was conducted [16]. Other blood and urine specimens (including for calcium-to-creatinine ratio) were shipped to the central laboratory at baseline and during follow up. Serum total 25(OH)D, which includes total 25(OH)D_3_ and total 25(OH)D_2_, from stored frozen fasting serum samples from the baseline and annual visits, was measured by liquid chromatography–tandem mass spectrometry with calibrators that are traceable to the National Bureau of Standards and Technology and validated by quarterly proficiency testing program administered by the Vitamin D External Quality Assessment scheme (DEQAS, United Kingdom) [17, 18]. The D2d study did not measure free 25(OH)D_3_ or free 25(OH)D_2_ levels.

### Statistical analyses

The sample size for the parent study was determined based on a target of 508 diabetes outcome events. The rationale has been previously published [5].

The frequency of AEs was evaluated in intention-to-treat analyses that compared groups defined by the randomization procedure and included all participants irrespective of adherence to assigned treatment or the protocol. All events were considered to occur independently, and no adjustment has been made for multiple events occurring in the same person. Incidence rates of AEs or differences in proportions of AEs were compared between the two groups. No adjustments were made for multiple comparisons.

The data analyses were generated using SAS software (Version 9.4 Copyright © 2019 SAS Institute Inc., Cary, NC, USA).

## Results

**Fig. 1:**
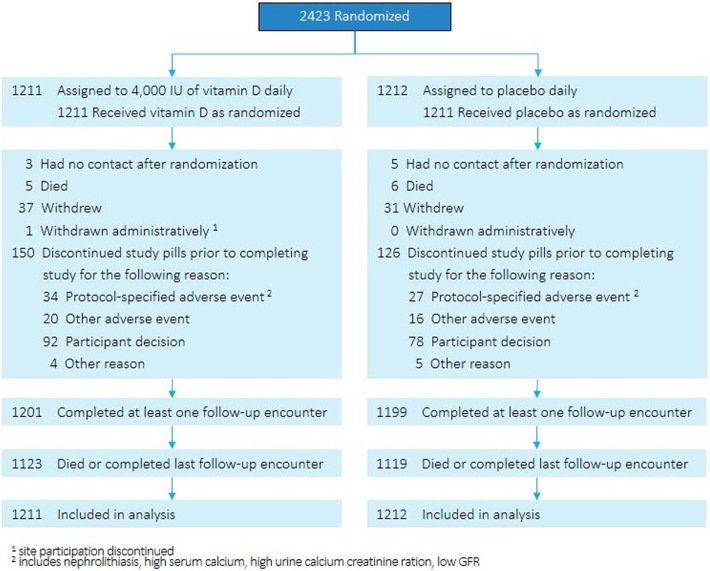
Flow of participants in the D2d Study. 2423 participants were randomized in D2d. 1211 were assigned to the vitamin D group and 1212 were assigned to the placebo group. Reasons for lost data or withdrawal from the study are presented as well as reasons for discontinuation of study pills by treatment assignment. 1201 participants in the vitamin D group completed at least one follow-up encounter and 1199 in the placebo group completed at least one follow-up encounter. 1123 participants in the vitamin D group died or completed last follow-up encounter and 1119 in the placebo group died or completed last follow-up encounter. 1211 in the vitamin D group and 1212 in the placebo group are included in these analyses.

From October 2013 through February 2017, 7133 people were screened, and 2423 were randomly assigned to vitamin D_3_ (*n* = 1211) or placebo (*n* = 1212), forming the intention-to-treat population (Fig. 1) [6]. Of those randomized, 45% of participants were women; 33% were of a non-White race; and 9% were of Hispanic ethnicity (Table 1) [19]. Mean age was 60.0 years; body-mass index, 32.1 kg/m^2^; and HbA1c, 5.9% (48 mmol/mol). Mean baseline serum 25(OH)D concentration was 28.0 ng/mL (68.8 nmol/L) with 78.3% of participants having a concentration equal to or greater than 20 ng/mL (49.1 nmol/L). There were no statistically significant differences in baseline characteristics by treatment assignment.

**Table 1:** Baseline characteristics of D2d participants^a^.

	Overall (*n* = 2423)	Vitamin D_3_ (*n* = 1211)	Placebo (*n* = 1212)
Characteristic			
Age, years	60.0 ± 9.9	59.6 ± 9.9	60.4 ± 10.0
Women, no. (%)	1086 (44.8)	541 (44.7)	545 (45.0)
Race, no. (%)^b^			
Asian	130 (5.4)	66 (5.5)	64 (5.3)
Black or African American	616 (25.4)	301 (24.9)	315 (26.0)
White	1616 (66.7)	810 (66.9)	806 (66.5)
Other	61 (2.5)	34 (2.8)	27 (2.3)
Hispanic or Latino Ethnicity, no. (%)^b^	225 (9.3)	120 (9.9)	105 (8.7)
Body-mass index, kg/m^2^	32.1 ± 4.5	32.0 ± 4.5	32.1 ± 4.4
Health history			
Medical conditions, no. (%)			
Hypercholesterolemia	1346 (55.6)	661 (54.6)	685 (56.5)
Cancer^c^	262 (10.8)	126 (10.4)	136 (11.2)
Cardiovascular disease^d^	305 (12.6)	106 (9.8)	199 (14.9)
Hypertension	1297 (53.5)	622 (51.4)	675 (55.7)
Dietary supplements^e^			
Vitamin D			
Participants taking vitamin D supplements, no. (%)	1037 (42.8)	508 (41.9)	529 (43.6)
Vitamin D intake among all participants, IU/day^f^	313 ± 398	310 ± 401	316 ± 397
Vitamin D intake among participants using supplements, IU/day	732 ± 254	739 ± 256	725 ± 253
Calcium			
Participants taking calcium supplements, no. (%)	804 (33.2)	385 (31.8)	419 (34.6)
Calcium intake among all participants, mg/day^f^	103 ± 176	100 ± 175	107 ± 176
Calcium intake among participants using supplements, mg/day	312 ± 167	316 ± 168	308 ± 166
Laboratory			
Serum 25-hydroxyvitamin D			
Mean, ng/mL	28.0 ± 10.2	27.7 ± 10.2	28.2 ± 10.1
Distribution, no. (%)			
<12 ng/mL	103 (4.3)	60 (5.0)	43 (3.6)
12–19 ng/mL	422 (17.4)	216 (17.8)	206 (17.0)
20–29 ng/mL	876 (36.2)	453 (37.4)	423 (34.9)
≥30 ng/mL	1021 (42.2)	482 (39.8)	539 (44.5)
Serum calcium, mg/dL	9.41 ± 0.37	9.40 ± 0.37	9.41 ± 0.38
Estimated glomerular filtration rate, mL/min/1.73 m^b, g^	87.1 ± 15.7	87.5 ± 15.6	86.7 ± 15.9
Fasting urine calcium-creatinine ratio	0.09 ± 0.06	0.09 ± 0.06	0.08 ± 0.06
Hematocrit, %^h^	42.8 ± 3.5	42.8 ± 3.4	42.8 ± 3.5

^a^Plus-minus values are means ± SD. Percentages may not add up to 100 because of rounding. To convert 25-hydroxyvitamin D from ng/mL to nmol/L, multiply by 2.456; to convert vitamin D intake from IU to mcg, divide by 40.

^b^Race and ethnicity were reported by the participant. The category “other” includes American Indian or Alaska Native; Native Hawaiian or other Pacific Islander; or other race. Ethnicity includes any race.

^c^Cancer (except for basal cell skin cancer) within 5 years of randomization was an exclusion criterion. Prostate cancer or well-differentiated thyroid cancer not expected to require treatment over the next 4 years were not exclusions. Persons with history of squamous cell cancer of the skin, which was completely excised and with no evidence of metastases, were eligible.

^d^Cardiovascular disease included: arrhythmias, chest pain, congestive heart failure, coronary artery disease, CABG/PCI, myocardial infarction, palpitations, peripheral vascular disease.

^e^Data on vitamin D and calcium intake are derived from a question about supplements, including multivitamins and high-dose prescribed doses. Participants were allowed to take, from supplements, up to 1000 IU/day of vitamin D and 600 mg/day of calcium. Dietary intake of vitamin D and calcium was not limited.

^f^Value shown is among all participants regardless of whether they reported use of supplements or not.

^g^Based on the Chronic Kidney Disease Epidemiology Collaboration equation.

^h^Individuals were excluded if they had anemia at screening visit defined as hematocrit <32% for women, <36% for men.

The last study encounter was in December 2018 and the trial was stopped when the number of prespecified diabetes events had occurred per protocol. The overall median follow-up was 3.0 years (vitamin D_3_ 3.0 [interquartile range, 2.0–3.6] years; placebo 2.9 [interquartile range, 2.0–3.5] years) and 99.1% of the cohort (1201 vitamin D_3_ and 1199 placebo group) contributed follow-up data.

The overall frequency of protocol-specified AEs of interest was low, with no significant between-group differences in the incidence of the first occurrence of the following protocol-specified adverse events of interest: hypercalcemia, hypercalciuria, hyperphosphatemia, low eGFR, metallic taste, fatigue / weakness, insomnia, polyuria, or nephrolithiasis (Table 2). There were 36 cases of participants with new-onset hypercalcemia on initial testing; on repeat testing, only 10 cases were confirmed, 6 in the vitamin D_3_ and 4 in the placebo group (incidence rate ratio [IRR] for vitamin D_3_ vs. placebo = 1.49; 95% CI 0.42, 5.27). There were 21 participants with new-onset hypercalciuria on initial testing; on repeat testing, only 2 cases were confirmed, 1 in each group (IRR for vitamin D_3_ vs. placebo = 0.99; 95% CI 0.06, 15.86). There were 3 cases of confirmed low eGFR, 1 in the vitamin D_3_ group and 2 in the placebo group (IRR for vitamin D_3_ vs. placebo = 0.50; 95% CI 0.04, 5.47). There were 52 self-reported cases of nephrolithiasis, 28 in the vitamin D_3_ and 24 in the placebo group (IRR for vitamin D_3_ vs. placebo = 1.16; 95% CI 0.67, 2.00) (Table 3). While the number of adverse events related to nausea/vomiting or poor appetite was low (*n* = 29), there were more cases reported among persons taking vitamin D_3_ compared to placebo, 20 vs 9 respectively (IRR for vitamin D_3_ vs. placebo = 2.20; 95% CI 1.00, 4.84). Supplementary Table 1 provides additional information on the total frequency of protocol-specified AEs of interest among persons who were taking their study pills at the time of the event.

**Table 2:** Frequency of first event of protocol-specified adverse events by group.

	Unique participants with first event during the study		
	Total (*n* = 2423)	Vitamin D_3_ (*n* = 1211)	Placebo (*n* = 1212)
Anemia	24	5	19
Fatigue and weakness	35	16	19
Headache	54	26	28
Hypercalcemia^a^	36	20	16
Hypercalcemia, confirmed^a^	10	6	4
Hypercalciuria^a^	21	11	10
Hypercalciuria, confirmed^a^	2	1	1
Hyperphosphatemia	0	0	0
Insomnia	32	13	19
Metallic taste	2	1	1
Nausea, vomiting, and/or poor appetite	29	20	9
Nephrolithiasis^b^	50	27	23
Low eGFR^a^	3	1	2
Low eGFR, confirmed	3	1	2
Polyuria	3	1	2

Adverse events were reported by participants (e.g., headache, insomnia) unless otherwise indicated (hypercalcemia, hypercalciuria, low eGFR).

Protocol-specified adverse event include those that have been previously associated with vitamin D (with / without calcium) administration (e.g., hypercalcemia) and other adverse events that may be of relevance due to intolerance to study pills (e.g., nausea).

^a^Based on in-study laboratory assessment. These adverse events may have required confirmation by repeat testing (“confirmed”), see text for details.

^b^Based on participant self-report and adjudicated by the Safety and Outcomes Subcommittee when medical records were available to review.

**Table 3:** Total adverse events through end of study by group.

	Vitamin D_3_ (*n* = 1211)		Placebo (*n* = 1212)		Incidence rate ratio with vitamin D (95% CI)
	Events	Events per 100 person-years	Events	Events per 100 person-years	
	no.		no.		
Adverse event	4039	116.1	4265	123.6	0.94 (0.90, 0.98)
Serious adverse event	260	7.47	269	7.80	0.96 (0.81, 1.14)
Death	5	0.14	6	0.17	0.83 (0.25, 2.71)
Hospitalization (new or prolongation)	250	7.18	264	7.65	0.94 (0.79, 1.12)
Any adverse event leading to discontinuation of study pills	58	1.67	46	1.33	1.25 (0.85, 1.84)
Within-study laboratory evaluation (confirmed with repeated testing)					
Hypercalcemia	6	0.17	4	0.12	1.49 (0.42, 5.27)
Hypercalciuria	1	0.03	1	0.03	0.99 (0.06, 15.86)
Low estimated glomerular filtration rate	1	0.03	2	0.06	0.50 (0.04, 5.47)
Self-reported					
Nephrolithiasis	28	0.80	24	0.70	1.16 (0.67, 2.00)

Hypercalcemia was defined as serum calcium (uncorrected for albumin concentration) higher than the upper limit of the normal range for the clinical laboratory at each clinical site; hypercalciuria was defined as fasting morning urine calcium-creatinine ratio over 0.375 measured by the central laboratory; low estimated glomerular filtration rate was defined as equal to or lower than 30 mL per min per 1.73 m^2^ of body-surface based on serum creatinine measured at each clinical site’s clinical laboratory using the Chronic Kidney Disease Epidemiology Collaboration equation. Unless a specific threshold was reached, hypercalcemia, hypercalciuria and low estimated glomerular filtration rate required confirmation (see “Methods”).

Table includes events in all participants who underwent randomization regardless of adherence; analyses censored at death, withdrawal, or end-of-study encounter (visit or phone call).

A total of 8304 AEs occurred during follow-up. The incidence rate of total AEs was lower in the vitamin D_3_ group (4039; 116.1 events per 100 person-years) compared to the placebo group (4265; 123.6 events per 100 person years) (IRR = 0.94; 95% CI 0.90, 0.98) (Table 3). A total of 529 SAEs occurred during follow-up. The incidence rate of SAEs was not different between the vitamin D_3_ (260 events; 7.47 per 100 person-years) and placebo groups (269; 7.80 per 100 person-years) (IRR = 0.96; 95% CI 0.81, 1.14). The majority of SAEs were for hospitalization and there was no statistically significant difference among the treatment groups (IRR = 0.94; 95% CI 0.79, 1.12) (Table 3). Supplementary Tables 2, 3, and 4 provide additional data for total AEs and SAEs by treatment group using an end organ classification. The vitamin D_3_ group had fewer AEs and SAEs for injury and musculoskeletal events.

Adherence to the intervention was high (84.1% of prescribed pills were taken) and a similar proportion of participants in the vitamin D_3_ group (12.1%) and placebo group (10.3%) stopped trial pills (difference, 1.8 percentage points; 95% CI, −0.7, 4.3). There was no significant difference between the proportions of participants who stopped study pills for any reason, including due to AEs or due to participant choice (17.5% in the vitamin D group vs. 16.0% in the placebo group). There was no significant difference between the proportions of participants who stopped study pills due to an AE: overall, 58 (4.8%) participants in the vitamin D group stopped trial pills due to an AE, including abnormal safety labs, compared to 46 (3.8%) in the placebo group (difference in proportions for vitamin D vs. placebo, 0.9% [95% CI, −0.6, 2.6%]) (Table 4).

**Table 4:** Trial pill tolerability by group.

Reason for permanent discontinuation of study pills, *n* ^a^	Overall (*n* = 2423)	Vitamin D_3_ (*n* = 1211)	Placebo (*n* = 1212)
Withdrew consent while on study pills	35	18	17
Adverse Event	89	50	39
Nephrolithiasis^b^	46	26	20
Other adverse event^c^	36	20	16
Death^d^	7	4	3
Safety labs—high serum calcium	10	6	4
Safety labs—high urine calcium	3	1	2
Safety labs— low GFR	2	1	1
Participant decision^e^	170	92	78
Other reason	9	4	5
Did not complete end-of-study encounter^f^	87	40	47

^a^Reasons for permanent discontinuation are *mutually exclusive*.

^b^Nephrolithiasis diagnosed by either a study physician or physician outside of D2d based on clinical, radiologic findings, or both. All cases—except 5—were classified as “possibly” or “probably related” to study pills.

^c^Other adverse event that led to discontinuation of study pills at the discretion of the site study physician or participant decision.

^d^Does not include 4 participants who stopped pills for another reason and died at a later date.

^e^A participant requested discontinuation of study pills for any other reason other than an adverse event.

^f^For participant who did not complete the end-of-study encounter, the study pill discontinuation date is the date of the last encounter that was completed.

## Discussion

In this multi‐center, randomized, double-blind, placebo‐controlled trial among overweight/obese persons at high risk of type 2 diabetes not selected for vitamin D insufficiency and who were screened and routinely monitored for safety, compared to placebo, oral vitamin D_3_ supplementation at a dose of 4000 IU per day (considered the UL for adults by the National Academy of Medicine) [13] was well-tolerated and did not result in an increased risk of AEs or SAEs, including side effects typically linked to vitamin D, such as hypercalcemia, nephrolithiasis, or hypercalciuria. This finding of no increased risk of AE or SAE in the vitamin D_3_ group is reassuring given that the majority of D2d participants began the trial with concentrations of serum 25(OH)D considered sufficient for healthy adults [6].

These data from the D2d study suggest that in similar populations of people who are overweight/obese and with prediabetes, the UL for safety and tolerability of vitamin D_3_ may be higher than previously established. The dose of vitamin D_3_ of 4000 IU per day used in the D2d study is the National Academy of Medicine recommended UL for persons over 8 years of age [13], and D2d participants were allowed to take up to 1000 IU of vitamin D_3_ on their own, for a maximum total dose of vitamin D of 5000 IU daily from supplements. The UL was established by the National Academy of Medicine in 2011 based on a synthesis of data indicating that a dose of 4000 IU was unlikely to cause hypercalcemia [20]. The National Academy of Medicine also chose a UL dose that would maintain a serum 25(OH)D concentration lower than 50–75 ng/mL (125–150 nmol/L), a concentration that was previously thought to be associated with adverse outcomes. However, the benefit-risk ratio may be different in populations that vary by BMI, skin complexion, or when a trial attempts to achieve higher 25(OH)D concentrations. Future trials are warranted to test the efficacy-safety ratio of higher doses of vitamin D supplementation that aim to achieve higher 25(OH)D levels in specific populations at-risk for specific conditions, such as diabetes, osteoporosis, cancer etc.

The dose of 4000 IU daily of vitamin D_3_ in D2d is a higher dose than administered in other recently completed clinical trials. For example, Lappe et al tested a 2000 IU daily dose of vitamin D_3_ and administered calcium supplements in addition to the vitamin D_3_ as part of the intervention arms in postmenopausal women [21, 22]; and the VITAL trial of adult men aged 50 or older and women aged 55 or older tested a dose of vitamin D_3_ 2000 IU daily without calcium [7]. Other trials include the New Zealand ViDA trial which tested a dose of vitamin D_3_ 100,000 IU taken monthly [23, 24]. Similar to the D2d study, these three trials have also reported few adverse events and no increased risk of hypercalcemia with vitamin D supplementation [7, 22, 23]. In contrast to the D2d study, only the Women’s Health Initiative Calcium / Vitamin D trial, with a much larger sample size and a longer follow-up period, reported an 17% increased risk of nephrolithiasis with combined daily 400 IU of vitamin D_3_ and 1000 mg of calcium compared to placebo (hazard ratio, 1.17; 95% CI, 1.02–1.34) [21].

There are trials with vitamin D supplementation that have reported other adverse events that were not assessed in the D2d study. A Canadian study found that, among healthy older adults (ages 55 to 70 years), vitamin D_3_ for 3 years at 4000 IU or 10,000 IU per day compared with 400 IU per day, resulted in statistically significant lower radial bone mineral density (BMD) as measured by high resolution peripheral quantitative computed tomography, but there were no significant differences in bone strength [25]. The D2d study did not assess BMD and thus cannot contribute information about the effect of the 4000 IU per day on BMD and bone strength in overweight/obese people with prediabetes. A trial conducted in Switzerland tested vitamin D_3_ at 60,000 IU per month vs. 24,000 IU per month and reported increased risk of falls with the higher dose [26]. The D2d study did not specifically query the participants regarding falls using a validated questionnaire; however the vitamin D_3_ had fewer AEs and SAEs for injury or musculoskeletal events than the placebo group (Supplementary Tables 2 and 4).

D2d is the first trial to assess the safety of vitamin D_3_ given at the tolerable upper intake level for adults by the National Academy of Medicine in a population of US adults with overweight/obesity and prediabetes. The D2d study has several additional strengths, including a large diverse group of participants who were at low risk for safety concerns related to vitamin D but at high risk for diabetes. The vitamin D_3_ dose of 4000 IU per day was selected to balance safety and efficacy and resulted in, on average, large differences in serum 25(OH)D concentrations between the vitamin D_3_ and placebo groups (54.3 ng/mL vs. 28.8 ng/mL, respectively, at month 24) [6]. Use of a placebo to blind investigators, staff, and participants to treatment assignment minimized ascertainment bias of adverse events, and careful attention to protocol fidelity resulted in a rigorously conducted clinical trial. Adverse events were collected frequently and in a similar way in both groups to reduce ascertainment bias. All serious adverse events and cases of nephrolithiasis were adjudicated by study investigators blinded to treatment assignment.

There are several considerations to put interpretation of our findings in context. As in all vitamin D trials, participants were excluded if they had a condition (e.g., high baseline serum calcium, etc.) that would have increased their risk for vitamin D-associated adverse events, and this may have reduced the occurrence of AEs compared to the general population taking vitamin D. The median time of follow-up in the D2d study was three years, and our findings may not extrapolate to longer term use of 4000 IU per day of vitamin D_3_. Thus, the risk of vitamin D associated AEs and SAEs may be greater among persons who are at higher risk, who take the supplement for longer periods of time, or who have been less carefully screened and monitored. Finally, the D2d study did not assess whether participants had a CYP24A1 mutation or other mutations in the vitamin D pathway, so we are unable to provide pharmacogenomic information.

## Conclusion

High-dose daily vitamin D_3_ supplementation at a dose of 4000 IU daily (considered the tolerable upper intake level for adults by the National Academy of Medicine) was safe and well tolerated among overweight/obese participants with prediabetes who were screened for risk of AEs and monitored for safety; and use of this supplement did not increase risk of AEs or SAEs, including side effects that have been previously associated with vitamin D.

## Supplementary information

Supplemental material file

## Data Availability

Datasets generated and analyzed during the current study and the associated data dictionary and code are not publicly available. Requests for datasets analyzed and code utilized in the current study can be made after acceptance for publication by bona fide researchers by submitting a request to the D2d Publications Committee. Individual participant data will be shared in a de-identified/anonymized format using a specialized SAS data platform. Protocol synopsis, contact details, publications, and the process for collaboration and data requests can be found on the website (d2dstudy.org).
