# Correction to: Safety and tolerability of high-dose daily vitamin D_3_ supplementation in the vitamin D and type 2 diabetes (D2d) study—a randomized trial in persons with prediabetes

**DOI:** 10.1038/s41430-022-01130-5

**Published:** 2022-04-13

**Authors:** Karen C. Johnson, Anastassios G. Pittas, Karen L. Margolis, Anne L. Peters, Lawrence S. Phillips, Ellen M. Vickery, Jason Nelson, Patricia R. Sheehan, David Reboussin, Saul Malozowski, Ranee Chatterjee, D2d research group, Anastassios G. Pittas, Irwin Brodsky, Lisa Ceglia, Chhavi Chadha, Ranee Chatterjee, Bess Dawson-Hughes, Cyrus Desouza, Rowena Dolor, John Foreyt, Adline Ghazi, Daniel S. Hsia, Karen C. Johnson, Sangeeta R. Kashyap, Sun Kim, Erin S. LeBlanc, Michael R. Lewis, Emilia Liao, Saul Malozowski, Lisa M. Neff, Patrick O’Neil, Jean Park, Anne Peters, Lawrence S. Phillips, Richard Pratley, Philip Raskin, Neda Rasouli, David Robbins, Clifford Rosen, Vanita R. Aroda, Patricia Sheehan, Myrlene A. Staten, James H. Ware, William C. Knowler

**Affiliations:** 1 Department of Preventive Medicine, University of Tennessee Health Science Center, Memphis, TN USA; 2 Division of Endocrinology, Diabetes, and Metabolism, Tufts Medical Center, Boston, MA USA; 3 HealthPartners Institute, Minneapolis, MN USA; 4 Keck School of Medicine, University of Southern California, Los Angeles, CA USA; 5 Atlanta VA Medical Center and Emory University School of Medicine, Decatur GA and Atlanta, GA USA; 6 Tufts Clinical and Translational Science Institute, Biostatistics, Epidemiology, and Research Design Center, Tufts Medical Center, Boston, MA USA; 7 Spaulding Rehabilitation Hospital, Boston, MA USA; 8 Department of Biostatistics and Data Science, Wake Forest School of Medicine, Winston-Salem, NC USA; 9 National Institute of Diabetes and Digestive and Kidney Disease, National Institutes of Health, Bethesda, MD USA; 10 Department of Medicine, Duke University School of Medicine, Durham, NC USA; 11 Tufts Medical Center, Boston, MA USA; 12 Maine Medical Center Research Institute, Scarborough, ME USA; 13 Tufts Medical Center, Boston, MA USA; 14 Health Partners Research Foundation, Minneapolis, MN USA; 15 Duke University Medical Center, Durham, NC USA; 16 Jean Mayer USDA Human Nutrition Research Center on Aging at Tufts University, Boston, MA USA; 17 Omaha VA Medical Center, University of Nebraska Medical Center, Omaha, NE USA; 18 Baylor College of Medicine, Houston, TX USA; 19 MedStar Good Samaritan Hospital, Baltimore, MD USA; 20 Pennington Biomedical Research Center, Baton Rouge, LA USA; 21 University of Tennessee Health Science Center, Memphis, TN USA; 22 Cleveland Clinic, Cleveland, OH USA; 23 Stanford University Medical Center, Stanford, CA USA; 24 Kaiser Permanente Center for Health Research NW, Portland, OR USA; 25 University of Vermont–Central Laboratory, Burlington, VT USA; 26 Northwell Health Lenox Hill Hospital, New York, NY USA; 27 National Institute of Diabetes and Digestive and Kidney Diseases, Bethesda, MD USA; 28 Northwestern Memorial Hospital, Chicago, IL USA; 29 Medical University of South Carolina, Charleston, SC USA; 30 MedStar Health Research Institute, Hyattsville, MD USA; 31 Keck School of Medicine of the University of Southern California, Los Angeles, CA USA; 32 Atlanta VA Medical Center, Decatur, GA and Emory University School of Medicine, Atlanta, GA USA; 33 AdventHealth Translational Research Institute for Metabolism and Diabetes, Orlando, FL USA; 34 University of Texas Southwestern Medical Center, Dallas, TX USA; 35 University of Colorado, School of Medicine and VA Eastern Colorado Health Care System, Aurora, CO USA; 36 University of Kansas Medical Center, Kansas City, KS USA; 37 Brigham and Women’s Hospital, Boston, MA USA; 38 Spaulding Rehabilitation Network, Boston, MA USA; 39 Harvard T.H. Chan School of Public Health, Boston, MA USA; 40 National Institute of Diabetes and Digestive and Kidney Diseases, Phoenix, AZ USA

**Keywords:** Nutrition, Metabolic disorders

Correction to: *European Journal of Clinical Nutrition* 10.1038/s41430-022-01068-8 published online 09 February 2022

The original version of the article contained some errors. Tables 1, 2, and 3 have been updated. Furthermore, the affiliation of the Past Steering Committee member James H. Ware was corrected. The original article has been corrected.

