# Supplementary material for: Safety and tolerability of high-dose daily vitamin D3 supplementation in the vitamin D and type 2 diabetes (D2d) study—a randomized trial in persons with prediabetes
Source: Eur J Clin Nutr. 2022 Feb 9;76(8):1117–24. doi: 10.1038/s41430-022-01068-8 (PMC9352576; doi:10.1038/s41430-022-01068-8)
Supplement: Supplementary file 1 — Supplemental material file [file 41430_2022_1068_MOESM1_ESM.docx]

**ONLINE SUPPORTING MATERIALS**

**Title:** Safety and Tolerability of High-Dose Daily Vitamin D_3_ Supplementation in the Vitamin D and Type 2 Diabetes Study - a Randomized Trial in Person with Prediabetes

**First Author:** Karen C. Johnson, MD, MPH

**Supplemental Table 1. Frequency of total events of protocol-specified AE^1^ by group among those on study pills at the time of the event**

|  | **Total number of events ^4^** | | |  |
| --- | --- | --- | --- | --- |
|  | **Total**  **(n=2,423)** | **Vitamin D_3_**  **(n=1211)** | **Placebo**  **(n=1212)** |  |
| Anemia | 26 | 5 | 21 |  |
| Fatigue and weakness | 37 | 16 | 21 |  |
| Headache | 60 | 29 | 31 |  |
| Hypercalcemia ^2^ | 39 | 22 | 17 |  |
| Hypercalcemia, confirmed^2^ | 10 | 6 | 4 |  |
| Hypercalciuria ^2^ | 23 | 13 | 10 |  |
| Hypercalciuria, confirmed^2^ | 2 | 1 | 1 |  |
| Hyperphosphatemia | 0 | 0 | 0 |  |
| Insomnia | 31 | 12 | 19 |  |
| Metallic taste | 2 | 1 | 1 |  |
| Nausea, vomiting, poor appetite | 30 | 20 | 10 |  |
| Nephrolithiasis ^3^ | 46 | 26 | 20 |  |
| Low eGFR ^2^ | 3 | 1 | 2 |  |
| Low eGFR, confirmed | 3 | 1 | 2 |  |
| Polyuria | 3 | 1 | 2 |  |

^1^Adverse events were reported by participants (e.g., headache, insomnia) unless otherwise indicated (hypercalcemia, hypercalciuria, low eGFR). Protocol-specified adverse event include those that have been previously associated with vitamin D (with / without calcium) administration (e.g., hypercalcemia) and other adverse events that may be of relevance due to intolerance to study pills (e.g., nausea).

^2^Based on in-study laboratory assessment. These adverse events may have required confirmation by repeat testing (“confirmed”), see text for details.

^3^ Based on participant self-report and adjudicated by the Safety and Outcomes Subcommittee when medical records were available to review.

^4^ Participant could have multiple reported instances of the same event**Supplemental Table 2. Total number of adverse events by group**

| **Event Organ Class** | **Overall**  **(n=2,423)** | **Vitamin D_3_**  **(n=1,211)** | **Placebo**  **(n=1,212)** |
| --- | --- | --- | --- |
| Total Adverse Events | 8304 | 4039 | 4265 |
| Blood and lymphatic system disorders | 47 | 13 | 34 |
| Cardiac disorders | 447 | 213 | 234 |
| Ear and labyrinth disorders | 186 | 98 | 88 |
| Endocrine disorders | 61 | 34 | 27 |
| Eye disorders | 232 | 117 | 115 |
| Gastrointestinal disorders | 688 | 336 | 352 |
| General disorders and administration site conditions | 262 | 136 | 126 |
| Hepatobiliary disorders | 46 | 28 | 18 |
| Immune system disorders | 98 | 41 | 57 |
| Infections and infestations | 209 | 106 | 103 |
| Injury, poisoning and procedural complications | 157 | 64 | 93 |
| Metabolism and nutrition disorders | 259 | 117 | 142 |
| Musculoskeletal and connective tissue disorders | 1746 | 859 | 887 |
| Neoplasm benign, malignant and unspecific (incl cysts and polyps) | 371 | 181 | 190 |
| Nervous system disorders | 421 | 207 | 214 |
| Psychiatric disorders | 82 | 46 | 36 |
| Renal and urinary disorders | 383 | 181 | 202 |
| Reproductive system and breast disorders | 102 | 49 | 53 |
| Respiratory, thoracic and mediastinal disorders | 1544 | 784 | 760 |
| Skin and subcutaneous tissue disorders | 503 | 237 | 266 |
| Surgical and medical procedures | 379 | 164 | 215 |
| Vascular disorders | 81 | 28 | 53 |

A participant may have contributed more than 1 event in each organ class. All events occurred post-randomization.

**Supplemental Table 3. Total number of participants with adverse events by group where organ class occurs in >5% of the entire cohort**

| **Event Organ Class** | **Overall**  **(n=2,423)** | **Vitamin D_3_**  **(n=1,211)** | **Placebo**  **(n=1,212)** |
| --- | --- | --- | --- |
| Total Adverse Events | 2011 | 1005 | 1006 |
| Cardiac disorders | 378 | 181 | 197 |
| Ear and labyrinth disorders | 157 | 80 | 77 |
| Eye disorders | 188 | 97 | 91 |
| Gastrointestinal disorders | 512 | 253 | 259 |
| General disorders and administration site conditions | 229 | 120 | 109 |
| Infections and infestations | 189 | 94 | 95 |
| Injury, poisoning and procedural complications | 132 | 59 | 73 |
| Metabolism and nutrition disorders | 244 | 112 | 132 |
| Musculoskeletal and connective tissue disorders | 1018 | 515 | 503 |
| Neoplasm benign, malignant and unspecific (incl cysts and polyps) | 305 | 153 | 152 |
| Nervous system disorders | 329 | 165 | 164 |
| Renal and urinary disorders | 301 | 146 | 155 |
| Respiratory, thoracic and mediastinal disorders | 890 | 455 | 435 |
| Skin and subcutaneous tissue disorders | 378 | 176 | 202 |
| Surgical and medical procedures | 293 | 133 | 160 |

A participant may have contributed more than 1 event in each organ class. All events occurred post-randomization.

**Supplemental Table 4. Total number of serious adverse events by group**

| **Event Organ Class** | **Overall**  **(n=2,423)** | **Vitamin D_3_**  **(n=1,211)** | **Placebo**  **(n=1,212)** |
| --- | --- | --- | --- |
| Total serious adverse events | 529 | 260 | 269 |
| Blood and lymphatic system disorders | 6 | 1 | 5 |
| Cardiac disorders | 71 | 40 | 31 |
| Ear and labyrinth disorders | 1 | 1 | 0 |
| Endocrine disorders | 5 | 2 | 3 |
| Eye disorders | 0 | 0 | 0 |
| Gastrointestinal disorders | 34 | 15 | 19 |
| General disorders and administration site conditions | 10 | 6 | 4 |
| Hepatobiliary disorders | 10 | 7 | 3 |
| Immune system disorders | 4 | 2 | 2 |
| Infections and infestations | 15 | 8 | 7 |
| Injury, poisoning and procedural complications | 15 | 6 | 9 |
| Metabolism and nutrition disorders | 2 | 1 | 1 |
| Musculoskeletal and connective tissue disorders | 21 | 7 | 14 |
| Neoplasm benign, malignant and unspecific (incl cysts and polyps) | 40 | 21 | 19 |
| Nervous system disorders | 33 | 21 | 12 |
| Psychiatric disorders | 3 | 2 | 1 |
| Renal and urinary disorders | 15 | 8 | 7 |
| Reproductive system and breast disorders | 1 | 0 | 1 |
| Respiratory, thoracic and mediastinal disorders | 43 | 24 | 19 |
| Skin and subcutaneous tissue disorders | 11 | 5 | 6 |
| Surgical and medical procedures | 177 | 79 | 98 |
| Vascular disorders | 12 | 4 | 8 |

A participant may have contributed more than 1 event in each organ class. All events are post-randomization.
